# Supplementary material for: Dual versus single vessel normothermic ex vivo perfusion of rat liver grafts using metamizole for vasodilatation
Source: PLoS One. 2020 Jul 2;15(7):e0235635. doi: 10.1371/journal.pone.0235635 (PMC7332079; doi:10.1371/journal.pone.0235635)
Supplement: S1 File — (DOCX) [file pone.0235635.s001.docx]

|  | **Dual Vessel \| Metamizole \| Pressure Control** | **Dual Vessel \| Metamizole \| Hourly Infusion** | **Dual Vessel \| w/o Metamizole** | **Single Vessel \| w/o Metamizole** | ***p*-Value** |
| --- | --- | --- | --- | --- | --- |
| **Rat Weight [g]** | 329 (37) | 335 (11.5) | 329.5 (13.5) | 318 (47.75) | 0.47 |
| **Liver Weight [g]** | 15.1 (1.33) | 17.35 (2.75) | 15.65 (1.2) | 14.8 (7.2) | 0.34 |
| **Cold Ischemic Time [min]** | 52.5 (14) | 55 (3.75) | 50 (10) | 55 (10) | 0.28 |
| **Macroscopic Flush** | 10 (0.75) | 10 (0.75) | 9.5 (1) | 10 (1) | 0.48 |
| **ALT [U/l]** |  |  |  |  |  |
| T0 | 30 (14.75) | 39 (17.75) | 39 (15) | 77 (84.25) | 0.04 |
| T3 | 88 (31.5) | 129.5 (69) | 110 (37.25) | 123.5 (210.5) | 0.07 |
| T6 | 193 (108.25) | 343.5 (343.25) | 372 (382) | 335.5 (485.5) | 0.22 |
| **AST [U/l]** |  |  |  |  |  |
| T0 | 53 (34.5) | 70.5 (42) | 77.5 (27.25) | 121 (121.25) | 0.31 |
| T3 | 196 (96.25) | 274.5 (232.25) | 310 (73.75) | 314 (306.25) | 0.16 |
| T6 | 459 (176.25) | 833.5 (754.25) | 927 (682) | 705 (1253.75) | 0.07 |
| **Urea [mmol/l]** |  |  |  |  |  |
| T0 | 9 (6.5) | 11.5 (5.25) | 11.5 (9.25) | 12 (10.25) | 0.87 |
| T3 | 36 (5.25) | 28.5 (9.75) | 26.5 (13) | 33.5 (20.5) | 0.26 |
| T6 | 44 (5) | 40 (8.75) | 37.5 (45.25) | 39 (24.75) | 0.74 |
| **Glucose [mg/dl]** |  |  |  |  |  |
| T0 | 320 (65.75) | 319 (146.5) | 351.5 (113.25) | 338 (225.75) | 0.81 |
| T3 | 250 (98.5) | 296 (86.5) | 242 (101.5) | 215 (141.5) | 0.31 |
| T6 | 256 (107.5) | 326 (58.5) | 230.5 (123.75) | 218 (137.5) | 019 |
| **Sodium [mmol/l]** |  |  |  |  |  |
| T0 | 136 (4.25) | 135.5 (3.25) | 136.5 (4.75) | 134 (3.75) | 0.75 |
| T3 | 135 (5.5) | 136 (7) | 132.5 (1.75) | 131.5 (1.75) | 0.04 |
| T6 | 137 (5.75) | 139.5 (10.5) | 132 (2.25) | 131.5 (4) | 0.01 |
| **Potassium [mmol/l]** |  |  |  |  |  |
| T0 | 4.9 (1.25) | 4.95 (1.4) | 5.35 (2.85) | 5 (1.33) | 0.93 |
| T3 | 3.9 (0.48) | 3.95 (0.33) | 4 (0.8) | 3.75 (0.8) | 0.77 |
| T6 | 4.6 (0.35) | 4.6 (0.3) | 4.55 (0.58) | 4.25 (1) | 0.52 |
| **Lactate [mg/dl]** |  |  |  |  |  |
| T0 | 27 (7.25) | 35 (3.75) | 31.5 (7) | 28.5 (30) | 0.67 |
| T3 | 14.5 (7) | 10 (8.5) | 15 (5) | 13.5 (7.75) | 0.43 |
| T6 | 7.5 (8.5) | 8 (5) | 12.5 (6.25) | 14 (7) | 0.07 |
| **Bile Production [mg]** |  |  |  |  |  |
| T1 | 672.5 (272) | 583.5 (152.5) | 628.5 (73.75) | 433.5 (281.75) | 0.4 |
| T2 | 541.5 (179.5) | 1021 (312.5) | 605 (175) | 498.5 (302.75) | 0.02 |
| T3 | 878.5 (682.75) | 1084.5 (119.5) | 586.5 (179.25) | 458 (106.5) | 0.02 |
| T4 | 803 (547.5) | 893.5 (219.5) | 501 (135.25) | 385 (130.75) | 0.006 |
| T5 | 766 (239.75) | 763 (230.5) | 400 (137) | 332 (67.75) | 0.008 |
| T6 | 773 (194.75) | 714 (277.75) | 362.5 (121.75) | 272 (32.75) | 0.006 |
| **pH** |  |  |  |  |  |
| T0 | 7.47 (0.13) | 7.5 (0.09) | 7.44 (0.12) | 7.4 (0.13) | 0.22 |
| T3 | 7.19 (0.15) | 7.2 (0.17) | 7.22 (0.11) | 7.3 (0.23) | 0.66 |
| T6 | 7.1 (0.1) | 7.15 (0.16) | 7.18 (0.21) | 7.23 (0.31) | 0.22 |
| **Oxygen Consumption [ml/min/g]** |  |  |  |  |  |
| T0 | 0.03 (0.02) | 0.03 (0.02) | 0.04 (0.01) | 0.02 (0.02) | 0.68 |
| T3 | 0.04 (0) | 0.04 (0) | 0.05 (0) | 0.04 (0.04) | 0.19 |
| T6 | 0.03 (0.01) | 0.05 (0.02) | 0.04 (0) | 0.03 (0.02) | 0.91 |
| **Portal Venous Pressure [mmHg]** |  |  |  |  |  |
| T0 | 5.55 (2.25) | 6.55 (2.8) | 4.45 (0.85) | 5.2 (3.85) | 0.26 |
| T1 | 4.4 (2.68) | 3.95 (2.8) | 4.45 (0.63) | 4.05 (1.68) | 0.83 |
| T2 | 4.95 (2.65) | 4.05 (3.1) | 4.7 (0.75) | 4.35 (1.43) | 0.74 |
| T3 | 4.8 (2.6) | 4.3 (2.73) | 5.6 (0.75) | 4.6 (1.65) | 0.63 |
| T4 | 4.85 (2.78) | 4.45 (2.73) | 5.55 (0.68) | 5.15 (1.65) | 0.64 |
| T5 | 4.3 (3.18) | 4.7 (2.98) | 5.45 (0.83) | 5.25 (1.9) | 0.71 |
| T6 | 4.65 (2.9) | 4.8 (3.3) | 5.2 (1.38) | 5.45 (1.68) | 0.87 |
| **Arterial Pressure [mmHg]** |  |  |  |  |  |
| T0 | 48.5 (9) | 49.5 (18.5) | 45 (18.75) | - | 0.67 |
| T1 | 43 (21.5) | 49 (23) | 48 (34.75) | - | 0.54 |
| T2 | 69.5 (54.5) | 554 (29.5) | 73 (18.5) | - | 0.78 |
| T3 | 90.5 (4.75) | 71.5 (22.5) | 140 (21.5) | - | 0.007 |
| T4 | 96.5 (25.75) | 83.5 (9.75) | 152 (26.75) | - | 0.01 |
| T5 | 91 (40.75) | 87.5 (22.5) | 160.5 (49.25) | - | 0.02 |
| T6 | 87.5 (29.25) | 85 (26.25) | 169.5 (24.75) | - | 0.03 |

|  | **Dual Vessel \| Metamizole \| Pressure Control** | **Dual Vessel \| Metamizole \| Hourly Infusion** | **Dual Vessel \| w/o Metamizole** | ***p*-Value** | ***Post hoc Test*** |
| --- | --- | --- | --- | --- | --- |
| **Rat Weight [g]** | 329 (37) | 335 (11.5) | 329.5 (13.5) | 0.58 |  |
| **Liver Weight [g]** | 15.1 (1.33) | 17.35 (2.75) | 15.65 (1.2) | 0.15 |  |
| **Cold Ischemic Time [min]** | 52.5 (14) | 55 (3.75) | 50 (10) | 0.17 |  |
| **Macroscopic Flush** | 10 (0.75) | 10 (0.75) | 9.5 (1) | 0.58 |  |
| **ALT [U/l]** |  |  |  |  |  |
| T0 | 30 (14.75) | 39 (17.75) | 39 (15) | 0.04 | 1 vs. 3 (p = 0.048) |
| T3 | 88 (31.5) | 129.5 (69) | 110 (37.25) | 0.09 |  |
| T6 | 193 (108.25) | 343.5 (343.25) | 372 (382) | 0.17 |  |
| **AST [U/l]** |  |  |  |  |  |
| T0 | 53 (34.5) | 70.5 (42) | 77.5 (27.25) | 0.39 |  |
| T3 | 196 (96.25) | 274.5 (232.25) | 310 (73.75) | 0.16 |  |
| T6 | 459 (176.25) | 833.5 (754.25) | 927 (682) | 0.07 |  |
| **Urea [mmol/l]** |  |  |  |  |  |
| T0 | 9 (6.5) | 11.5 (5.25) | 11.5 (9.25) | 0.79 |  |
| T3 | 36 (5.25) | 28.5 (9.75) | 26.5 (13) | 0.1 |  |
| T6 | 44 (5) | 40 (8.75) | 37.5 (45.25) | 0.32 |  |
| **Glucose [mg/dl]** |  |  |  |  |  |
| T0 | 320 (65.75) | 319 (146.5) | 351.5 (113.25) | 0.55 |  |
| T3 | 250 (98.5) | 296 (86.5) | 242 (101.5) | 0.31 |  |
| T6 | 256 (107.5) | 326 (58.5) | 230.5 (123.75) | 0.17 |  |
| **Sodium [mmol/l]** |  |  |  |  |  |
| T0 | 136 (4.25) | 135.5 (3.25) | 136.5 (4.75) | 0.79 |  |
| T3 | 135 (5.5) | 136 (7) | 132.5 (1.75) | 0.11 |  |
| T6 | 137 (5.75) | 139.5 (10.5) | 132 (2.25) | 0.02 | 2 vs. 3 (0.04) |
| **Potassium [mmol/l]** |  |  |  |  |  |
| T0 | 4.9 (1.25) | 4.95 (1.4) | 5.35 (2.85) | 0.81 |  |
| T3 | 3.9 (0.48) | 3.95 (0.33) | 4 (0.8) | 0.9 |  |
| T6 | 4.6 (0.35) | 4.6 (0.3) | 4.55 (0.58) | 0.86 |  |
| **Lactate [mg/dl]** |  |  |  |  |  |
| T0 | 27 (7.25) | 35 (3.75) | 31.5 (7) | 0.25 |  |
| T3 | 14.5 (7) | 10 (8.5) | 15 (5) | 0.3 |  |
| T6 | 7.5 (8.5) | 8 (5) | 12.5 (6.25) | 0.14 |  |
| **Bile Production [mg]** |  |  |  |  |  |
| T1 | 672.5 (272) | 583.5 (152.5) | 628.5 (73.75) | 0.58 |  |
| T2 | 541.5 (179.5) | 1021 (312.5) | 605 (175) | 0.02 | 1 vs. 2 (0.02) |
| T3 | 878.5 (682.75) | 1084.5 (119.5) | 586.5 (179.25) | 0.06 |  |
| T4 | 803 (547.5) | 893.5 (219.5) | 501 (135.25) | 0.02 | 2 vs. 3 (0.04) |
| T5 | 766 (239.75) | 763 (230.5) | 400 (137) | 0.02 | 1 vs. 3 (0.04) |
| T6 | 773 (194.75) | 714 (277.75) | 362.5 (121.75) | 0.02 | 1 vs. 3 (0.03) |
| **pH** |  |  |  |  |  |
| T0 | 7.47 (0.13) | 7.5 (0.09) | 7.44 (0.12) | 0.23 |  |
| T3 | 7.19 (0.15) | 7.2 (0.17) | 7.22 (0.11) | 0.78 |  |
| T6 | 7.1 (0.1) | 7.15 (0.16) | 7.18 (0.21) | 0.14 |  |
| **Portal Venous Pressure [mmHg]** |  |  |  |  |  |
| T0 | 5.55 (2.25) | 6.55 (2.8) | 4.45 (0.85) | 0.09 |  |
| T1 | 4.4 (2.68) | 3.95 (2.8) | 4.45 (0.63) | 0.78 |  |
| T2 | 4.95 (2.65) | 4.05 (3.1) | 4.7 (0.75) | 0.69 |  |
| T3 | 4.8 (2.6) | 4.3 (2.73) | 5.6 (0.75) | 0.49 |  |
| T4 | 4.85 (2.78) | 4.45 (2.73) | 5.55 (0.68) | 0.6 |  |
| T5 | 4.3 (3.18) | 4.7 (2.98) | 5.45 (0.83) | 0.51 |  |
| T6 | 4.65 (2.9) | 4.8 (3.3) | 5.2 (1.38) | 0.76 |  |
| **Arterial Pressure [mmHg]** |  |  |  |  |  |
| T0 | 48.5 (9) | 49.5 (18.5) | 45 (18.75) | 0.67 |  |
| T1 | 43 (21.5) | 49 (23) | 48 (34.75) | 0.54 |  |
| T2 | 69.5 (54.5) | 54 (29.5) | 73 (18.5) | 0.78 |  |
| T3 | 90.5 (4.75) | 71.5 (22.5) | 140 (21.5) | 0.007 | 2 vs. 3 (p = 0.005) |
| T4 | 96.5 (25.75) | 83.5 (9.75) | 152 (26.75) | 0.01 | 2 vs. 3 (p = 0.01) |
| T5 | 91 (40.75) | 87.5 (22.5) | 160.5 (49.25) | 0.02 | 2 vs. 3 (p = 0.03) |
| T6 | 87.5 (29.25) | 85 (26.25) | 169.5 (24.75) | 0.03 | - |

|  | **Dual Vessel \| Metamizole \| Pressure Control** | **Single Vessel \| w/o Metamizole** | ***p*-Value** |
| --- | --- | --- | --- |
| **Rat Weight [g]** | 329 (37) | 318 (47.75) | 0.69 |
| **Liver Weight [g]** | 15.1 (1.33) | 14.8 (7.2) | 1.0 |
| **Cold Ischemic Time [min]** | 52.5 (14) | 55 (10) | 0.49 |
| **Macroscopic Flush** | 10 (0.75) | 10 (1) | 0.69 |
| **ALT [U/l]** |  |  |  |
| T0 | 30 (14.75) | 77 (84.25) | 0.06 |
| T3 | 88 (31.5) | 123.5 (210.5) | 0.03 |
| T6 | 193 (108.25) | 335.5 (485.5) | 0.11 |
| **AST [U/l]** |  |  |  |
| T0 | 53 (34.5) | 121 (121.25) | 0.11 |
| T3 | 196 (96.25) | 314 (306.25) | 0.06 |
| T6 | 459 (176.25) | 705 (1253.75) | 0.03 |
| **Urea [mmol/l]** |  |  |  |
| T0 | 9 (6.5) | 12 (10.25) | 0.49 |
| T3 | 36 (5.25) | 33.5 (20.5) | 0.89 |
| T6 | 44 (5) | 39 (24.75) | 0.89 |
| **Glucose [mg/dl]** |  |  |  |
| T0 | 320 (65.75) | 338 (225.75) | 0.89 |
| T3 | 250 (98.5) | 215 (141.5) | 0.49 |
| T6 | 256 (107.5) | 218 (137.5) | 0.69 |
| **Sodium [mmol/l]** |  |  |  |
| T0 | 136 (4.25) | 134 (3.75) | 0.34 |
| T3 | 135 (5.5) | 131.5 (1.75) | 0.11 |
| T6 | 137 (5.75) | 131.5 (4) | 0.03 |
| **Potassium [mmol/l]** |  |  |  |
| T0 | 4.9 (1.25) | 5 (1.33) | 0.89 |
| T3 | 3.9 (0.48) | 3.75 (0.8) | 0.69 |
| T6 | 4.6 (0.35) | 4.25 (1) | 0.34 |
| **Lactate [mg/dl]** |  |  |  |
| T0 | 27 (7.25) | 28.5 (30) | 1.0 |
| T3 | 14.5 (7) | 13.5 (7.75) | 0.69 |
| T6 | 7.5 (8.5) | 14 (7) | 0.11 |
| **Bile Production [mg]** |  |  |  |
| T1 | 672.5 (272) | 433.5 (281.75) | 0.2 |
| T2 | 541.5 (179.5) | 498.5 (302.75) | 0.29 |
| T3 | 878.5 (682.75) | 458 (106.5) | 0.11 |
| T4 | 803 (547.5) | 385 (130.75) | 0.03 |
| T5 | 766 (239.75) | 332 (67.75) | 0.03 |
| T6 | 773 (194.75) | 272 (32.75) | 0.03 |
| **pH** |  |  |  |
| T0 | 7.47 (0.13) | 7.4 (0.13) | 0.2 |
| T3 | 7.19 (0.15) | 7.3 (0.23) | 0.49 |
| T6 | 7.1 (0.1) | 7.23 (0.31) | 0.2 |
| **Portal Venous Pressure [mmHg]** |  |  |  |
| T0 | 5.55 (2.25) | 5.2 (3.85) | 0.89 |
| T1 | 4.4 (2.68) | 4.05 (1.68) | 1.0 |
| T2 | 4.95 (2.65) | 4.35 (1.43) | 0.69 |
| T3 | 4.8 (2.6) | 4.6 (1.65) | 0.69 |
| T4 | 4.85 (2.78) | 5.15 (1.65) | 0.69 |
| T5 | 4.3 (3.18) | 5.25 (1.9) | 0.49 |
| T6 | 4.65 (2.9) | 5.45 (1.68) | 0.69 |

|  | **Dual Vessel \| Metamizole \| Pressure Control** | **Dual Vessel \| w/o Metamizole** | **Single Vessel \| w/o Metamizole** | **Dual Vessel \| Metamizole \| Hourly Infusion** | ***p*-Value** |
| --- | --- | --- | --- | --- | --- |
| **LDH** |  |  |  |  |  |
| T1 | 33.45 (10.53) | 21.85 (22.18) | 45.85 (88.5) | 27.5 (14.4) | 0.11 |
| T2 | 31.65 (18.33) | 25.85 (19.3) | 40.75 (68.65) | 53.5 (40.08) | 0.05 |
| T3 | 80.6 (60.28) | 63 (27.6) | 63.3 (84.15) | 66.35 (54.6) | 0.88 |
| T4 | 87.35 (122.8) | 116 (43.5) | 94.9 (141.5) | 76.85 (72.23) | 0.76 |
| T5 | 127.5 (113.5) | 242.65 (82.63) | 203.35 (479.5) | 85.8 (84.53) | 0.08 |
| T6 | 148.6 (93.63) | 420.95 (202.55) | 234.9 (76-470.3) | 96.25 (96.75) | 0.05 |
| **yGT** |  |  |  |  |  |
| T1 | 910.4 (492.47) | 965.95 (741.4) | 897.3 (477.55) | 816.8 (167.6) | 0.73 |
| T2 | 544.05 (168.97) | 724.3 (236.92) | 578.45 (605.72) | 151.6 (77.8) | 0.03 |
| T3 | 518.45 (665.15) | 1286.4 (704.05) | 978.4 (821.15) | 321 (290.25) | 0.03 |
| T4 | 722.7 (975.33) | 1534.5 (1498.65) | 1402.6 (877.05) | 518.4 (641.55) | 0.06 |
| T5 | 1005.25 (992.9) | 1983.1 (3848.2) | 2030.6 (2384.15) | 10349.01 (612.6) | 0.07 |
| T6 | 1117.15 (2325.98) | 2982 (3042.2) | 2392.7 (2280.45) | 887.7 (491.5) | 0.04 |
| **Necrosis** | 0 | 2 (3.5) | 2.25 (4.63) | 1 (5.75) | 0.36 |
| **Sinusoidal Dilat.** | 10 (0) | 15 (32.5) | 30 (30) | 20 (20) | 0.05 |
| **TUNEL+** | 0.03 (0.07) | 0.26 (0.44) | 0.22 (0.55) | 0.11 (0.71) | 0.07 |

|  | **Dual Vessel \| Metamizole \| Pressure Control** | **Single Vessel \| w/o Metamizole** | ***p*-Value** |
| --- | --- | --- | --- |
| **LDH** |  |  |  |
| T1 | 33.45 (10.53) | 45.85 (88.5) | 0.11 |
| T2 | 31.65 (18.33) | 40.75 (68.65) | 0.34 |
| T3 | 80.6 (60.28) | 63.3 (84.15) | 1 |
| T4 | 87.35 (122.8) | 94.9 (141.5) | 0.69 |
| T5 | 127.5 (113.5) | 203.35 (479.5) | 0.34 |
| T6 | 148.6 (93.63) | 234.9 (76-470.3) | 0.4 |
| **yGT** |  |  |  |
| T1 | 910.4 (492.47) | 897.3 (477.55) | 0.89 |
| T2 | 544.05 (168.97) | 578.45 (605.72) | 1.0 |
| T3 | 518.45 (665.15) | 978.4 (821.15) | 0.34 |
| T4 | 722.7 (975.33) | 1402.6 (877.05) | 0.34 |
| T5 | 1005.25 (992.9) | 2030.6 (2384.15) | 0.2 |
| T6 | 1117.15 (2325.98) | 2392.7 (2280.45) | 0.2 |
| **Necrosis** | 0 | 2.25 (4.63) | 0.02 |
| **Sinusoidal Dilat.** | 11 (3.75) | 30 (30 | < 0.001 |
| **TUNEL+** | 0.03 (0.07) | 0.22 (0.55) | 0.03 |

|  | **Dual Vessel \| Metamizole \| Pressure Control** | **Dual Vessel \| w/o Metamizole** | **Dual Vessel \| Metamizole \| Hourly Infusion** | ***p*-Value** | ***Post hoc Test*** |
| --- | --- | --- | --- | --- | --- |
| **LDH** |  |  |  |  |  |
| T1 | 33.45 (10.53) | 21.85 (22.18) | 27.5 (14.4) | 0.33 |  |
| T2 | 31.65 (18.33) | 25.85 (19.3) | 53.5 (40.08) | 0.02 | 2 vs. 3 p = 0.02 |
| T3 | 80.6 (60.28) | 63 (27.6) | 66.35 (54.6) | 0.60 |  |
| T4 | 87.35 (122.8) | 116 (43.5) | 76.85 (72.23) | 0.58 |  |
| T5 | 127.5 (113.5) | 242.65 (82.63) | 85.8 (84.53) | 0.04 | 2 vs. 3 p = 0.04 |
| T6 | 148.6 (93.63) | 420.95 (202.55) | 96.25 (96.75) | 0.02 | 2 vs. 3 p = 0.04 |
| **yGT** |  |  |  |  |  |
| T1 | 910.4 (492.47) | 965.95 (741.4) | 816.8 (167.6) | 0.5 |  |
| T2 | 544.05 (168.97) | 724.3 (236.92) | 151.6 (77.8) | 0.01 | 2 vs. 3 p = 0.01 |
| T3 | 518.45 (665.15) | 1286.4 (704.05) | 321 (290.25) | 0.04 | 2 vs. 3 p = 0.03 |
| T4 | 722.7 (975.33) | 1534.5 (1498.65) | 518.4 (641.55) | 0.04 | 2 vs. 3 p = 0.04 |
| T5 | 1005.25 (992.9) | 1983.1 (3848.2) | 10349.01 (612.6) | 0.08 |  |
| T6 | 1117.15 (2325.98) | 2982 (3042.2) | 887.7 (491.5) | 0.049 | 2 vs. 3 p = 0.04 |
| **Necrosis** | 0 | 2 (3.5) | 1 (5.75) | 0.09 | - |
| **Sinusoidal Dilat.** | 10 (0) | 15 (32.5) | 20 (20) | 0.01 | 1 vs. 3 p = 0.01 |
| **TUNEL+** | 0.03 (0.07) | 0.26 (0.44) | 0.11 (0.71) | 0.06 |  |

LDH two way ANOVA

| Tukey's multiple comparisons test | Mean Diff. | 95.00% CI of diff. | Significant? | Summary | Adjusted P Value |
| --- | --- | --- | --- | --- | --- |
|  |  |  |  |  |  |
| 1 |  |  |  |  |  |
| Dual Vessel \| w/o Metamizol vs. Dual Vessel \| Metamizol \| Hourly Infusion | -3.975 | -79.46 to 71.51 | No | ns | 0.9912 |
| Dual Vessel \| w/o Metamizol vs. Dual Vessel \| Metamizol \| Pressure Control | -6.45 | -81.93 to 69.03 | No | ns | 0.9769 |
| Dual Vessel \| Metamizol \| Hourly Infusion vs. Dual Vessel \| Metamizol \| Pressure Control | -2.475 | -77.96 to 73.01 | No | ns | 0.9966 |
|  |  |  |  |  |  |
| 2 |  |  |  |  |  |
| Dual Vessel \| w/o Metamizol vs. Dual Vessel \| Metamizol \| Hourly Infusion | -36.78 | -112.3 to 38.71 | No | ns | 0.4736 |
| Dual Vessel \| w/o Metamizol vs. Dual Vessel \| Metamizol \| Pressure Control | -5.875 | -81.36 to 69.61 | No | ns | 0.9808 |
| Dual Vessel \| Metamizol \| Hourly Infusion vs. Dual Vessel \| Metamizol \| Pressure Control | 30.9 | -44.58 to 106.4 | No | ns | 0.5884 |
|  |  |  |  |  |  |
| 3 |  |  |  |  |  |
| Dual Vessel \| w/o Metamizol vs. Dual Vessel \| Metamizol \| Hourly Infusion | -16.65 | -92.13 to 58.83 | No | ns | 0.8562 |
| Dual Vessel \| w/o Metamizol vs. Dual Vessel \| Metamizol \| Pressure Control | -16.68 | -92.16 to 58.81 | No | ns | 0.8558 |
| Dual Vessel \| Metamizol \| Hourly Infusion vs. Dual Vessel \| Metamizol \| Pressure Control | -0.025 | -75.51 to 75.46 | No | ns | >0.9999 |
|  |  |  |  |  |  |
| 4 |  |  |  |  |  |
| Dual Vessel \| w/o Metamizol vs. Dual Vessel \| Metamizol \| Hourly Infusion | 28.03 | -47.46 to 103.5 | No | ns | 0.6459 |
| Dual Vessel \| w/o Metamizol vs. Dual Vessel \| Metamizol \| Pressure Control | 13.25 | -62.23 to 88.73 | No | ns | 0.9062 |
| Dual Vessel \| Metamizol \| Hourly Infusion vs. Dual Vessel \| Metamizol \| Pressure Control | -14.78 | -90.26 to 60.71 | No | ns | 0.8848 |
|  |  |  |  |  |  |
| 5 |  |  |  |  |  |
| Dual Vessel \| w/o Metamizol vs. Dual Vessel \| Metamizol \| Hourly Infusion | 141.2 | 65.67 to 216.6 | Yes | *** | 0.0001 |
| Dual Vessel \| w/o Metamizol vs. Dual Vessel \| Metamizol \| Pressure Control | 113.6 | 38.09 to 189.1 | Yes | ** | 0.0018 |
| Dual Vessel \| Metamizol \| Hourly Infusion vs. Dual Vessel \| Metamizol \| Pressure Control | -27.58 | -103.1 to 47.91 | No | ns | 0.6549 |
|  |  |  |  |  |  |
| 6 |  |  |  |  |  |
| Dual Vessel \| w/o Metamizol vs. Dual Vessel \| Metamizol \| Hourly Infusion | 324.2 | 248.7 to 399.7 | Yes | **** | <0.0001 |
| Dual Vessel \| w/o Metamizol vs. Dual Vessel \| Metamizol \| Pressure Control | 310.5 | 235 to 386 | Yes | **** | <0.0001 |
| Dual Vessel \| Metamizol \| Hourly Infusion vs. Dual Vessel \| Metamizol \| Pressure Control | -13.73 | -89.21 to 61.76 | No | ns | 0.8998 |

yGT two way ANOVA

| Tukey's multiple comparisons test | Mean Diff. | 95.00% CI of diff. | Significant? | Summary | Adjusted P Value |
| --- | --- | --- | --- | --- | --- |
|  |  |  |  |  |  |
| 1 |  |  |  |  |  |
| Dual Vessel \| w/o Metamizol vs. Dual Vessel \| Metamizol \| Hourly Infusion | 14.22 | -30.95 to 59.38 | No | ns | 0.7298 |
| Dual Vessel \| w/o Metamizol vs. Dual Vessel \| Metamizol \| Pressure Control | -0.4475 | -45.61 to 44.72 | No | ns | 0.9997 |
| Dual Vessel \| Metamizol \| Hourly Infusion vs. Dual Vessel \| Metamizol \| Pressure Control | -14.66 | -59.83 to 30.5 | No | ns | 0.7154 |
|  |  |  |  |  |  |
| 2 |  |  |  |  |  |
| Dual Vessel \| w/o Metamizol vs. Dual Vessel \| Metamizol \| Hourly Infusion | 34.02 | -11.15 to 79.19 | No | ns | 0.1743 |
| Dual Vessel \| w/o Metamizol vs. Dual Vessel \| Metamizol \| Pressure Control | -17 | -62.17 to 28.17 | No | ns | 0.6382 |
| Dual Vessel \| Metamizol \| Hourly Infusion vs. Dual Vessel \| Metamizol \| Pressure Control | -51.02 | -96.19 to -5.854 | Yes | * | 0.0233 |
|  |  |  |  |  |  |
| 3 |  |  |  |  |  |
| Dual Vessel \| w/o Metamizol vs. Dual Vessel \| Metamizol \| Hourly Infusion | 45.43 | 0.2591 to 90.59 | Yes | * | 0.0484 |
| Dual Vessel \| w/o Metamizol vs. Dual Vessel \| Metamizol \| Pressure Control | 28.4 | -16.77 to 73.56 | No | ns | 0.2919 |
| Dual Vessel \| Metamizol \| Hourly Infusion vs. Dual Vessel \| Metamizol \| Pressure Control | -17.03 | -62.2 to 28.14 | No | ns | 0.6372 |
|  |  |  |  |  |  |
| 4 |  |  |  |  |  |
| Dual Vessel \| w/o Metamizol vs. Dual Vessel \| Metamizol \| Hourly Infusion | 4.745 | -40.42 to 49.91 | No | ns | 0.9653 |
| Dual Vessel \| w/o Metamizol vs. Dual Vessel \| Metamizol \| Pressure Control | 7.445 | -37.72 to 52.61 | No | ns | 0.9168 |
| Dual Vessel \| Metamizol \| Hourly Infusion vs. Dual Vessel \| Metamizol \| Pressure Control | 2.7 | -42.47 to 47.87 | No | ns | 0.9886 |
|  |  |  |  |  |  |
| 5 |  |  |  |  |  |
| Dual Vessel \| w/o Metamizol vs. Dual Vessel \| Metamizol \| Hourly Infusion | 50.04 | 4.869 to 95.2 | Yes | * | 0.0266 |
| Dual Vessel \| w/o Metamizol vs. Dual Vessel \| Metamizol \| Pressure Control | 16.71 | -28.45 to 61.88 | No | ns | 0.6478 |
| Dual Vessel \| Metamizol \| Hourly Infusion vs. Dual Vessel \| Metamizol \| Pressure Control | -33.32 | -78.49 to 11.84 | No | ns | 0.1866 |
|  |  |  |  |  |  |
| 6 |  |  |  |  |  |
| Dual Vessel \| w/o Metamizol vs. Dual Vessel \| Metamizol \| Hourly Infusion | 29.1 | -16.07 to 74.26 | No | ns | 0.2750 |
| Dual Vessel \| w/o Metamizol vs. Dual Vessel \| Metamizol \| Pressure Control | -6.628 | -51.79 to 38.54 | No | ns | 0.9335 |
| Dual Vessel \| Metamizol \| Hourly Infusion vs. Dual Vessel \| Metamizol \| Pressure Control | -35.73 | -80.89 to 9.441 | No | ns | 0.1467 |
