# Supplementary material for: Dual versus single vessel normothermic ex vivo perfusion of rat liver grafts using metamizole for vasodilatation
Source: PLoS One. 2020 Jul 2;15(7):e0235635. doi: 10.1371/journal.pone.0235635 (PMC7332079; doi:10.1371/journal.pone.0235635)
Supplement: Attachment — Submitted filename: Response to Reviewers.docx [file pone.0235635.s003.docx]

**PLOS ONE**

Dr. Joerg Heber

Editor-in-Chief

**Revised Manuscript PONE-D-20-06139**

**Dual versus single vessel normothermic *ex vivo* perfusion of rat liver grafts using metamizole for vasodilatation.**

Felix Claussen, Joseph M.G.V. Gassner, Simon Moosburner, David Wyrwal, Maximilian Nösser, Peter Tang, Lara Wegener, Julian Pohl, Anja Reutzel-Selke, Ruza Arsenic, Johann Pratschke, Igor M. Sauer, Nathanael Raschzok

Dear Dr. Heber,

Thank you very much for giving us the opportunity to submit a newly revised version of our manuscript. We hereby submit our revised manuscript as clean copy (Manuscript.docx) and additional copy with the changes being done highlighted (Revised Manuscript with Track Changes.docx). Please find below our specific answers to the remaining questions and suggestions raised by Reviewer #3 as a point-to-point response. We hope that our manuscript is appropriate for publication in PLOS ONE.

Yours sincerely,

**Reviewer #3**

*I have two minor comments: First, the question re. ATP from two reviewers was not addressed. I do appreciate that performing such analyses may require repeat of most if not all experiments which in my opinion is not necessary and would likely be unnecessary duplication that would not justify the use of research animals; one reason the Mergenthal criteria was recommended as an alternative to ATP is it can be evaluated practically. Since the authors have already done this in the revision, I would have recommended the authors to note this in their rebuttal so it does not appear like an ignored comment to the reviewers.*

Thank you for pointing this out. We apologize for the negligence.

*My second comment would be if the authors could shed some light on if they noticed any difference in relation to edema (or lack thereof) between the groups? Noting that pressures in dNEVLP in the metamizole on-demand group were better, I would be interested to see if this correlates in the context of edema as well.*

Thank you for your remark and we apologize for not addressing this aspect in our first rebuttal. The assessment of edema in our work was limited to the histopathological examination of the livers. The pathologist did not find relevant edema in any of the four groups. We now mention this in the manuscript (“Results” part, p. 13*: “Histopathology did not show relevant edema in any of the four groups after perfusion.”* and “Discussion” part, p. 19: “*Interestingly, the high arterial pressures did not lead to relevant development of edema*.”)

The assessment of edema by lyophilisation is currently being established in our workgroup. Preliminary results correlate with the results from our histopathological examination and do not show significant edema after neither single vessel nor dual vessel perfusion when compared to native livers. These results indicate that the development of edema is not a relevant problem in our small animal NEVLP model. However, we will further investigate this aspect in our future projects and thank you for your constructive feedback.
